# Identifying pathways for large-scale implementation of a school-based mental health programme in the Eastern Mediterranean Region: a theory-driven approach

**DOI:** 10.1093/heapol/czaa124

**Published:** 2020-11-06

**Authors:** Olakunle Alonge, Anna Chiumento, Hesham M Hamoda, Eman Gaber, Zill-e- Huma, Maryam Abbasinejad, Walaa Hosny, Alia Shakiba, Ayesha Minhas, Khalid Saeed, Lawrence Wissow, Atif Rahman

**Affiliations:** 1 Department of International Health, Johns Hopkins Bloomberg School of Public Health, 615 N Wolfe Street, E8140, Baltimore, MD 21205, USA; 2 Department of Psychological Sciences, Institute of Population Health Sciences, The University of Liverpool, Block B, Waterhouse Buildings, 1-5 Brownlow Street, Liverpool L69 3GL, UK; 3 Department of Psychiatry, Boston Children’s Hospital 300 Longwood Avenue, Boston, MA 02115, USA; 4 General Secretariat of Mental Health and Addiction Treatment, Ministry of Health, Al-Inshaa WA Al-Munirah, El-Sayeda Zainab, Cairo Governorate, Egypt; 5 Human Development Research Foundation, House 06, Street 55, F-7/4, Islamabad, Pakistan; 6 Department for Mental Health and Substance Abuse, Ministry of Health and Medical Education, Shahrak-e-Gharb, Eivanak Blvd, Islamic Republic of Iran; 7 Department of Psychiatry, Tehran University of Medical Sciences, Tehran, Islamic Republic of Iran; 8 Institute of Psychiatry, Benazir Bhutto Hospital, Benazir Bhutto Road, Chah Sultan, Rawalpindi, Punjab 46000, Pakistan; 9 Department of Non-communicable Diseases and Mental Health, World Health Organization, Regional Office for the Eastern Mediterranean, Monazamet El Seha El Alamia Street, Nasr City, Cairo 11371, Egypt; 10 Department of Psychiatry and Behavioral Sciences, Division of Child and Adolescent Psychiatry, University of Washington, 1959 NE Pacific Street, Seattle WA 98195, USA

**Keywords:** School mental health, implementation, Eastern Mediterranean Region, theory of change

## Abstract

Globally there is a substantial burden of mental health problems among children and adolescents. Task-shifting/task-sharing mental health services to non-specialists, e.g. teachers in school settings, provide a unique opportunity for the implementation of mental health interventions at scale in low- and middle-income countries (LMICs). There is scant information to guide the large-scale implementation of school-based mental health programme in LMICs. This article describes pathways for large-scale implementation of a School Mental Health Program (SMHP) in the Eastern Mediterranean Region (EMR). A collaborative learning group (CLG) comprising stakeholders involved in implementing the SMHP including policymakers, programme managers and researchers from EMR countries was established. Participants in the CLG applied the theory of change (ToC) methodology to identify sets of preconditions, assumptions and hypothesized pathways for improving the mental health outcomes of school-aged children in public schools through implementation of the SMHP. The proposed pathways were then validated through multiple regional and national ToC workshops held between January 2017 and September 2019, as the SMHP was being rolled out in three EMR countries: Egypt, Pakistan and Iran. Preconditions, strategies and programmatic/contextual adaptations that apply across these three countries were drawn from qualitative narrative summaries of programme implementation processes and facilitated discussions during biannual CLG meetings. The ToC for large-scale implementation of the SMHP in the EMR suggests that identifying national champions, formulating dedicated cross-sectoral (including the health and education sector) implementation teams, sustained policy advocacy and stakeholders engagement across multiple levels, and effective co-ordination among education and health systems especially at the local level are among the critical factors for large-scale programme implementation. The pathways described in this paper are useful for facilitating effective implementation of the SMHP at scale and provide a theory-based framework for evaluating the SMHP and similar programmes in the EMR and other LMICs.

**KEY MESSAGES**

- Despite a huge burden of mental health problems among children and adolescents globally, there is insufficient information to guide large-scale implementation of population-based mental health interventions like the School Mental Health Programme (SMHP).
- A regional collaborative learning group (CLG) comprising of a range of stakeholders from countries of Eastern Mediterranean Region (EMR) and using the theory of change (ToC) methodology identified pathways and strategies to implement SMHP at scale.
- There are subtle differences between large-scale implementation and scale-up of school-based mental health interventions. Continuous advocacy at policy levels, ongoing stakeholders’ engagement and cross-sectoral implementation teams is a key for successful implementation across diverse country systems and settings.
- The CLG and ToC approaches facilitated local ownership of the programme and stakeholders’ engagement in child mental health broadly; and bridged the implementation knowledge gap between regional policy representatives and national programme implementers.

## Introduction

Globally, an estimated 10–20% of children and adolescents are affected by mental health problems, and 90% of these live in low- and middle-income countries (LMICs) (Kieling *et al.*, 2011). Studies have shown that common mental health problems including anxiety, behavioural and mood disorders begin to manifest before 14 years of age in most contexts (Merikangas *et al.*, 2010). Approximately, 60% of the population of the Eastern Mediterranean Region (EMR) are 19 years or younger and 10–36% of this population experience mental health problems (World Health Organisation, 2011), with depressive and anxiety disorders being the most common (Charara *et al.*, 2017). Furthermore, children exposed to conflict and other humanitarian emergencies prevalent within the EMR have been found to experience higher rates of mental health problems (Attanayake *et al.*, 2009; Reed *et al.*, 2012) which give rise to the potential need for additional specialized services when providing targeted mental health interventions (Jordans *et al.*, 2009; Farooq *et al.*, 2015).

The EMR has a gross deficit of mental health resources for young people across all sectors (Rahman *et al.*, 2019). In 2017, the median number of mental health workers (encompassing psychiatrists, child psychiatrists, other medical doctors, nurses, psychologists, occupational therapists, social workers and other related professionals working in mental health) in the Eastern Mediterranean was 7.7 per 100 000 population (World Health Organisation, 2018). Globally, there are <0.1 child psychiatrists for every 100 000 population across all LMICs (World Health Organisation, 2018). Furthermore, for 78 countries that provided data, <9% of mental health workers provided child and adolescent mental health services (World Health Organisation, 2018). This lack of human resources for mental health globally and in the EMR further hampers efforts to provide mental health services to children and adolescents.

Several authors have noted that a life-course approach is required to address the mental health problems experienced by children and adolescents, recommending integrating services into existing social, educational and health systems (Collins *et al.*, 2011; Benningfield and Stephan, 2015; Murphy *et al.*, 2017; Patel *et al.*, 2018). School-based programmes offer particular opportunities for the prevention, early identification and management of mental health problems among children (Forman *et al.*, 2009; Langley *et al.*, 2010; Stallard *et al.*, 2014; Das *et al.*, 2016; Murphy *et al.* 2017). These include the accessibility of the school setting, the reduced stigma associated with receiving mental health services in schools, opportunities for parental engagement, and the integration of mental health support into routine education. Other studies have shown that the incorporation of task-shifting approaches—where some of the tasks performed by mental health specialists e.g. psychiatrists are ‘shifted’ to non-specialists e.g. teachers who receive targeted training and supervision from mental health specialists to guide them in performing these tasks—are efficient and effective for delivering mental health services in resource-limited settings like the EMR, and with children (Murray *et al.*, 2011; Patel *et al.*, 2018; Purgato *et al.*, 2018).

Recognizing this context, child mental health has been identified as a priority in the World Health Organization’s (WHO) Eastern Mediterranean Regional Framework for Mental Health [hereafter ‘Regional Framework’] (Gater *et al.*, 2015) which contextualizes the WHO’s Comprehensive Mental Health Action Plan 2013–20 (WHO Resolution WHA66/8) to the EMR (Saxena *et al.*, 2015). The prioritization of child mental health in the Regional Framework encapsulates intervention development, implementation and research to support the identification of effective pathways for scaling-up evidence-based interventions to the national level (Gater *et al.*, 2015). Based on the life-course approach and the principle of task-shifting, the WHO EMR Office developed an evidence-based manualized School Mental Health Programme (SMHP) to address child and adolescent mental health problems in the region. The manual provides guidelines for universal and targeted interventions for addressing common emotional and behavioural problems including depression, anxiety, suicidal thoughts, attention deficits and post-traumatic reactions; and is tailored to be provided by non-specialists, including teachers, administrators, school nurses, social workers and school counsellors (Supplementary File S1) (World Health Organisation Office for the Eastern Mediterranean Region, 2014; Imran *et al.*, 2018). The SMHP is implemented through cascade trainings where master trainers (child and adolescent mental health specialists) train national/district trainers, who themselves cascade this training to nominated school staff; with ongoing supervision of non-specialists provided by national/district trainers throughout the process of SMHP training and delivery (Murray *et al.*, 2011).

The SMHP is a complex intervention combining multiple strategies that operate at various levels. Like other complex interventions or multifaceted strategies, the risk of failure is high without a clear understanding of how it may work in a particular setting, and of the implementation activities and resources to support its effective delivery (Peters *et al.*, 2009). Although there are currently multiple randomized controlled trials under way evaluating the effectiveness of the SMHP (Imran *et al.*, 2018; ClinicalTrials.gov, 2019), there is insufficient information that could guide its large-scale implementation globally, including in the EMR. This gap in understanding the implementation pathways for the SMHP, and theory of how the SMHP works under real-world conditions limits its large-scale impact and integration into existing health and education systems in LMICs, despite efforts to promote its widespread adoption by the WHO Eastern Mediterranean Regional Office (EMRO). Countries adopting the SMHP need to know how the programme works in order to adapt it to their own context; and also understand the set of actors, implementation activities and resources required for implementing the SMHP at scale. Such understanding would inform theory-based evaluation of adapted SMHP programmes in various settings, and innovative strategies to further reduce the high burden of mental health problems among school-aged children in the EMR, and LMICs more broadly.

Established in 2016 and with support from the WHO EMRO, the School Health Implementation Network (SHINE) was created to enhance co-operation and collaboration among practitioners, researchers and policymakers responsible for implementing and evaluating the SMHP in EMR countries. The network includes partners in Egypt, Iran, Jordan and Pakistan; with support from academic facilitators from the University of Liverpool in the UK, and Harvard and the Johns Hopkins Universities in the USA. A core objective of the SHINE network is to support participating countries in the contextual adaptation, implementation and evaluation of the SMHP, generating country and regional-level evidence about its effectiveness and implementation strategies across country and health system contexts.

The objective of this paper is to describe the SHINE network’s collaborative process for developing the pathways used at the regional and individual country levels for large-scale implementation of the SMHP. These processes are based on theories developed by SHINE partners and their initial experiences implementing the SMHP in the EMR. It is hoped that these theories will provide a roadmap for how to implement complex mental health interventions at scale in LMICs.

## Methods

The method for developing pathways for large-scale implementation of the SMHP involved four main stages.

### Stage 1

We convened a collaborative learning group (CLG) of participants from the SHINE partner countries (Egypt, Iran, Jordan and Pakistan) to facilitate peer-to-peer learning among countries implementing SMHP in the EMR. CLGs usually consist of peers working together to complete a task, solve a problem or create a product (Laal and Ghodsi 2012). CLG members achieve improved understanding of the activities involved in the task, problem or product through mutual co-operation and consensus building. The SHINE CLG included policymakers from the Ministries of Health (MoH), programme managers from national non-governmental organizations (NGOs) and mental health practitioners and academic researchers working to implement SMHP in Egypt, Iran, Jordan and Pakistan. The CLG members meet in-person for 3–5 days biannually to discuss progress with the SMHP implementation activities in their respective countries, and hold frequent online meetings in between the in-person meetings.

### Stage 2

The CLG conducted an initial regional theory of change (ToC) workshop on pathways for implementing the SMHP at scale to achieve improved mental health clinical outcomes among school-aged children in the EMR countries involved. A ToC outlines the relationships among a set of preconditions or outcomes that must be fulfilled before a programme goal can be achieved, and describes a logical sequence through which a programme works to achieve a given goal. It makes explicit the assumptions under which the outcomes are obtained, and the contextual factors that influence the relationships among these outcomes (Vogel, 2012; Breuer *et al.*, 2016). The SHINE regional ToC workshop included two participants directly involved with planning for the SMHP implementation in each CLG country, including policymakers from the MoH, programme managers from national NGOs and mental health practitioners and academic researchers. Two mental health experts with WHO EMRO affiliation and significant experience conducting child mental health activities also participated. The regional ToC was developed over a 2-day workshop held in Cairo, Egypt in May 2017 and was facilitated by an expert in implementation science. The first step in developing the regional ToC was to identify a common goal for the SMHP which was acceptable to all workshop participants, agreed as: the improvement of mental health clinical outcomes among school-aged children in the EMR. Next, workshop participants were asked to list all necessary preconditions for attaining the common SMHP goal (e.g. political buy-in at all relevant policy levels, increased resources to address mental health issues in schools), based on their in-country experiences and knowledge working in the health and educational sectors, and any initial planning activities for the SMHP implementation. These were then assessed by a consensus process (i.e. a majority of participants have to accept that a listed precondition is necessary), and the nature and sequence of relationships among these preconditions were agreed. Where there were disagreements on a precondition, the issue was put to a vote and the facilitator’s vote was used to break any deadlock. A regional ToC map was then developed by the facilitator by compiling the prioritized preconditions in the order that was agreed upon at the workshop to illustrate these preconditions and organize the relationships among them. Next, key assumptions about what needs to be in place for the ToC pathways to occur were assessed by the participants. These assumptions relate to the structure, climate and governance of the broader health and educational systems in the countries involved, e.g. availability of teachers that can be trained, existence of a tiered health services delivery system that can facilitate referrals for specialized child mental health services.

### Stage 3

The regional ToC map was validated by CLG participants with in-country partners through a series of national ToC workshops and meetings. The methods for conducting the national ToC workshops and meetings varied by country (Table 1), and were conducted to confirm the relationships described in the regional ToC map with national and subnational stakeholders including policymakers at various ministries, mental health services providers, teachers and school administrators. Furthermore, the workshops and meetings were conducted to note country-specific contextual and intervention adaptations that were necessary when the regional ToC was applied to individual countries. These adaptations (and their rationale) were captured using concepts from the Consolidated Framework for Implementation Research (CFIR) (Damschroder *et al.*, 2009) and were summarized and compared for the different countries during another regional ToC workshop within the CLG in February 2019. The CFIR concepts that were used to describe the adaptive changes include characteristics of the implementers, organizations involved, process of implementation (planning, engaging, executing and evaluating) and the broader external context (political and socioeconomic environment) (Supplementary File S2).

**Table 1:** Summary of SMPH ToC development process in the EMR

	Regional TOC development at CLG workshops	National TOC validation exercises		
		Egypt	Pakistan	Iran
No of Workshops/Meetings	2 workshops	2 workshops 4 lectures 10 meetings	2 workshops	1 workshop 17 meetings
Time period	May 2017 to September 2019	March 2017 to June 2019	November 2016 to April 2017	September 2018 to April 2019
Composition of participant involved	MOH: 3 MOE: 1 WHO: 2 Academics: 3 Others^a^: 4	MOH: 29 MOE: 5 Lecture attendees: ∼50 Others^a^: 21	MOH: 7 MOE: 22 Others**: 15	MOH: 7 MOE: 24 Academics: 6 Others**: 3
Convener of the TOC exercise	SHINE network CLG facilitators	General Secretariat of Mental Health and Substance Abuse, National Ministry of Health, Egypt	The Institute of Psychiatry, National Ministry of Health, Pakistan in collaboration with Human Development Research Foundation (an NGO)	The National School Mental Health Team, Ministry of Health, Iran
Key method	Stakeholder meeting	Individual and group stakeholder meetings	Stakeholder meetings	Focus groups
Key procedure at the TOC	Free listing, ranking, voting, review and feedback	Free listing, ranking, voting, review and feedback	Free listing, ranking, review and feedback	Free listing

a Others include NGO representatives, psychologists, and representatives from other ministries and government agencies.

### Stage 4

Further validation of the relationships described in the ToC was conducted based on experiences of SMHP implementation. The CLG participants from Egypt, Iran and Pakistan had initiated an initial SMHP implementation around the same time as national ToC workshops and meetings were being conducted in these countries. At the CLG bi-annual meetings between February 2017 and September 2019, representatives from each country provided a narrative summary of the activities conducted in the previous 6 months, the contextual and intervention adaptations that occurred, and any specific strategies that were used to achieve preconditions outlined in the regional ToC map. A facilitated discussion was conducted following the presentation of the narrative summaries to clarify the relationships between the different preconditions within each country context. Based on the narrative summaries and meeting notes from the facilitated discussions, the hypothesized pathways from the ToC were revisited focusing on the key preconditions, adaptations and strategies to describe the actual implementation pathway for the delivery of SMHP across the different countries. The strategies included in the implementation pathway also draw from a review of published literature on large-scale school mental health programmes mostly from high-income countries (HICs) (Murphy *et al.*, 2017), including the Promoting Alternative Thinking Strategies (PATHS) , Cognitive-Behavioural Intervention for Trauma in Schools (CBITS) (Stein *et al.*, 2003), Positive Behavioural Intervention and Supports (PBIS) (Bradshaw *et al.*, 2010), Classroom-based Cognitive Behavioural Therapy (FRIENDS) (Stallard *et al.*, 2014), Positive Action (Li *et al*., 2011), Skills for Life (Guzman *et al.*, 2015) and Mind Matters (Wyn *et al*., 2000). Given that most of the SHINE countries were in their early phase of implementation, the pathway described below is relevant to the early phase of large-scale implementation of the SMHP.

## Results

**Figure 1:**
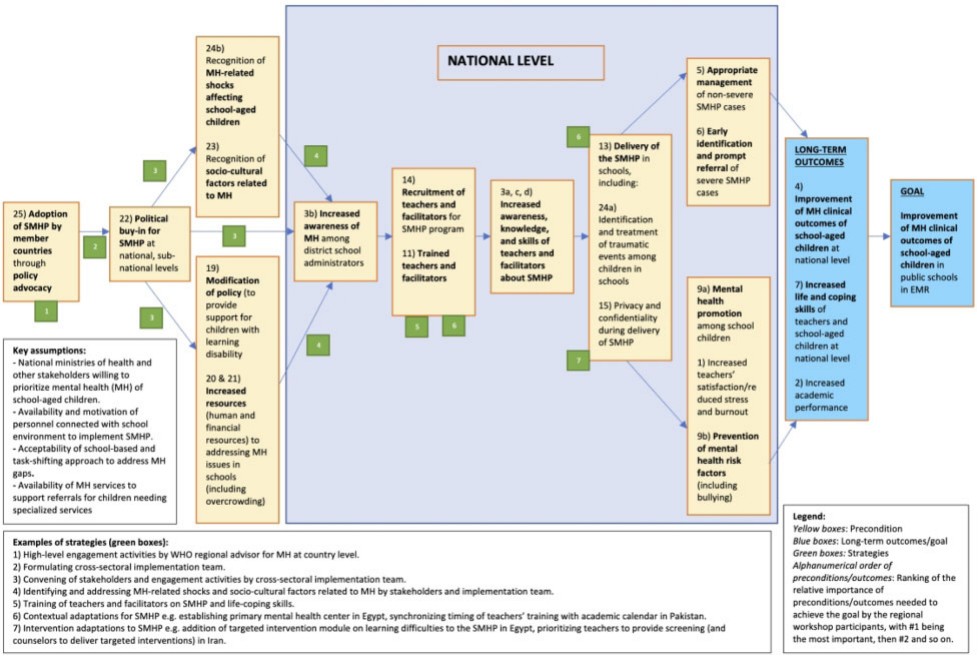
Regional ToC for the implementation of the SMHP in the EMR.

The regional ToC map and the hypothesized implementation pathway for the SMHP in the EMR developed during the regional ToC workshop (Stage 2) is described in Figure 1. This map represents the set of preconditions, relationships and key assumptions that all stakeholders agreed were necessary for the large-scale implementation of the SMHP across Egypt, Iran and Pakistan.

As of September 2019, over 300 public school teachers have been trained or involved in the early-phase implementation of the SMHP across the EMR. The SMHP is being implemented in 6 and 27 public schools in Iran and Egypt, respectively; and has reached 72 public schools in Pakistan. Based on the initial implementation activities and feedback from in-country meetings and workshops (Stages 3 and 4), the implementation pathway for improving the mental health outcomes of school-aged children in public schools using the SMHP in the EMR rests upon several key preconditions which overlap with the preconditions described in the ToC map. First, a core team of national champions that facilitate planning, engagement and advocacy with relevant stakeholders needs to be identified. This national team targets key ministries including education and health, and relevant NGOs at the outset and over the course of implementation. Such advocacy is conducted at national and sub-national levels for large-scale implementation and to facilitate the institutionalization of implementation processes. Key advocacy outcomes are to secure the buy-in of policy-makers and administrators; promote accommodations to school policy and practice that favour integrating the SMHP into educational systems; facilitate resources needed for implementing interventions included in the SMHP; promote collaborative exchanges such as referral pathways between the education and health systems; and raise awareness of key social determinants relevant for promoting the mental health of school-aged children. Experience from the EMR suggests that such policy advocacy is initially based on informal networks of the national team of champions, opportunistic engagements with policymakers, support from the WHO EMRO and advocacy by WHO representatives with key stakeholders such as the secretaries/ministers of health.

Second, continuous policy advocacy and engagements at national and sub-national levels were identified as crucial for SMHP implementation. This precondition was differentially prioritized in the countries involved based on the subtle differences in the SMHP implementation in each country. Large-scale implementation of complex interventions such as the SMHP (the implementation goal of the SMHP in Egypt and Iran), and going to scale with the SMHP after an initial effectiveness research study (the implementation goal of the SMHP in Pakistan) are related, but not equivalent, objectives. Large-scale implementation starts with the intention to get the programme to as many eligible units (schools in this case) as possible from the outset of the programme and learn along the process; while going to scale after an initial research study aims first to establish the feasibility and scalability of the programme among limited and selected eligible units, before later establishing a process for scaling-up the programme to other eligible units. The implementation pathway developed through the SHINE partnership outlines steps for both the scale-up and large-scale implementation of the SMHP across multiple countries. However, continuous policy advocacy and engagement as a precondition are especially prioritized for large-scale implementation where active strategies to integrate the intervention into existing systems are identified and deployed from the point of programme initiation. Whilst continuous advocacy and engagement are also required for facilitating an initial study with the hope of future scale-up, the intensity and scale of advocacy are much bigger and varied, and becomes a major project goal under large-scale implementation as opposed to being subordinated to helping a study achieve its aims before future scale-up. The sustained policy advocacy for large-scale implementation requires extensive resources throughout the SMHP implementation process, as well as approaches to seek a sustainable re-allocation of resources from within existing systems.

Third, the national team of champions also forms a central pillar of the SMHP implementation team, responsible for planning and engaging with other implementers, executing the implementation plan and facilitating the monitoring and evaluation of implementation activities in each country. The full implementation team, in conjunction with key administrators within the health and educational systems, is responsible for identifying national trainers and for supporting the cascade of training to teachers and allied school staff. The national trainers may differ between contexts and may include Ministry of Health or Education personnel or health professionals including psychiatrists and psychologists. It is important that the national trainers are connected to both the education and health sectors, especially primary mental health services, as they are the first point of referral for children needing specialized mental health care. Where no primary mental health services exist, the pathway to large-scale impact requires establishing this primary level of care and associated referral pathways into secondary and tertiary care to sufficiently address the increased demand for specialized mental health services that is predicted to result from the SMHP implementation.

Fourth, cross-sectoral collaboration, including power-sharing and co-decision-making between the MoH and Ministries of Education (MoE), is crucial for the large-scale implementation and impact of the SMHP. Both of these ministries often have existing mechanisms for providing health services to school-aged children, with large-scale implementation of the SMHP calling for harmonization and integration where parallel mechanisms exist. This precondition is perhaps the most challenging to achieve, especially in contexts where ministries are siloed and there is limited inter-ministerial collaborations at the system level. The national team of champions originates from the Ministry of Health for the SMHP implementation in the EMR and plays a significant role in breaking such silos by consciously reaching across the divide, aiming to expand the full implementation team to include key individuals within the Ministry of Education. Such outreach requires compromise and a willingness to share power and seek joint ownership of the SMHP, including any resulting outcomes.

Fifth, local champions are needed at the schools, education districts and at the interface between schools and primary health care at the community level. These local champions may include personnel at the cascade level, including NGO managers, local school and health administrators, psychology graduates without clinical training, counsellors or champion teachers depending on the setting. They work alongside national trainers to ensure fidelity of the implementation process, and to advocate for mental health promotion with parents and community members via community forums and parent–teacher interactions. Key outcomes of the activities of these local champions are increased awareness, knowledge and skills to address child mental health problems among schoolteachers and community members, which aims to contribute to destigmatizing mental health problems within schools and at the community level.

**Figure 2:**
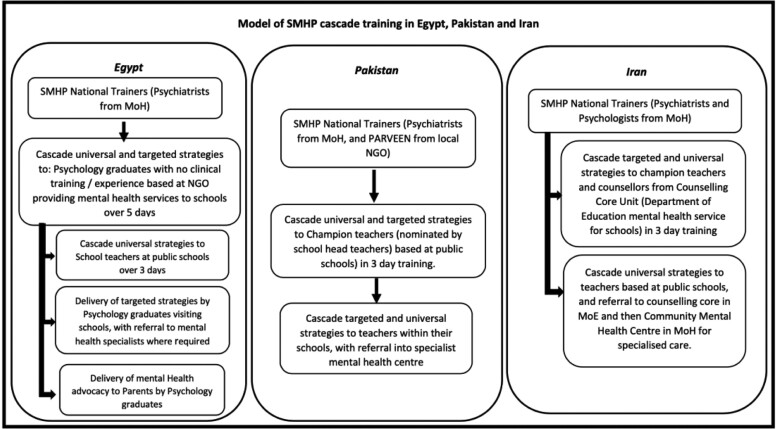
Model of SMHP cascade training and referral pathways in Egypt, Pakistan and Iran.

Last, the delivery of the SMHP involves the provision of either the universal intervention only, or both the universal and targeted mental health interventions, by different cadres of teachers and personnel within the schools, depending on the resources available at country/district levels, i.e. different schools have different categories of personnel who performed similar roles in implementing the SMHP (see Figure 2). The delivery requires explicit protocols for providing mental health promotion, prevention and care to school children, teacher self-care, detailed supervisory mechanisms and well-delineated referral pathways for specialist mental health care to ensure that the interventions and delivery strategies contribute directly to the improvement of mental health outcomes among school-aged children. The referral pathways across all countries include teachers identifying children who need additional mental health support beyond the classroom setting and referring them to champion teachers, psychologists or counsellors who then either provide services or refer them to primary/secondary mental health services within the country’s health system. Strain on the existing mental health services occurs in some places, and this manifests as incomplete referrals to primary/secondary mental health services due to limited capacity of the system to absorb the increased demand for services or barriers to access such as parents facing out-of-pocket costs for services.

Key assumptions for the described preconditions include the willingness of MoH personnel and other stakeholders to prioritize the mental health of school-aged children; the availability and strong motivation of personnel connected with school environment (teachers, psychology graduate and school counsellors) to implement the SMHP; the acceptability of a school-based and task-shifting approach to address gaps in mental health services by parents, teachers and policy-makers; and the availability of referral pathways for children in need of specialized services. These assumptions are consistent across the different EMR country contexts where the SMHP is being implemented.

Table 2 below summarizes the key strategies, contextual and intervention adaptations conducted to facilitate the preconditions described above, and that are specific to each country context, along with the rationale for these strategies and adaptations.

**Table 2:** Country-specific strategies and adaptations for the implementation of SMHP in EMR

ToC factors	Egypt	Pakistan	Iran
Key strategies^a^	1. Formulation of implementation team within an existing agency (General Secretariat of Mental Health and Addiction Treatment) of the ministry of health (MoH) with historical collaboration with the Department of Environmental, Population and Health Education of the ministry of education (MoE). *Rationale:* The strategic position of the implementation team within MoH and straddling MoH-MoE will facilitate cross-collaboration between MoE and MoH needed for the scale-up of the programme. 2. Inclusion of NGOs (e.g. Save the Children) who are already working in promoting child health at scale with external funds in the implementation team. *Rationale*: This will facilitate increased resources for the initial rollout and adoption of the programme.	1. Selection of a government tertiary health institution to lead large-scale implementation. *Rationale:* The MOH and MOE are both well-represented in the tertiary health institution—and this will facilitate cross-sectoral collaboration among these two ministries. The tertiary health institution also has existing capacity in training, and historical collaboration with schools and primary health-care systems. 2. Signing of an official memorandum of understanding (MOU) between the MOE and MOH. *Rationale*: The initial MOU provides a legitimate framework for support and collaboration among different sub- agencies under these respective ministries for implementation. It is anticipated that additional MOUs will be required in future.	1. High-level negotiations between the MoH and MoE which preserved the role of the MoE as the principal agent responsible for programme activities at the school level, and MoH providing supervisory support to the schools, and oversight at other levels, including the referral pathways for specialized services. *Rationale:* The high-level negotiation among the two ministries led to compromise and collaboration without which the SMHP programme would not have been implemented.
Contextual adaptation^b^	1. Elevating the role of school psychologists (instead of teachers) to deliver targeted interventions. They also provide training to teachers, serve as supervisors, and as first point of referral for specialized care. *Rationale:* The professional background of school psychologists and their role in the educational system makes them more suitable in delivering the SMHP. The training and skills that they received from MoH trainers are strong motivating factors, which also solidifies their placement within the school system. 2. Establishment of a primary mental health centre where the school psychologists engage directly with parents and community members (and also provide targeted mental health interventions with support of psychiatrists from other levels of care). *Rationale:* The primary mental health centre will facilitate the psychologist’s activities at the school and serve as a referral centre for identified cases, linking the school system with the health services delivery system. The primary mental health centre provides efficiency in that it increases the reach of the limited number psychologists, rather than having some schools without psychologists. 3. Engaging with the nascent national health insurance scheme to incorporate the scale-up (and funding) for SMHP as a preventative health strategy for mental health at the primary health-care level. *Rationale:* The national health insurance scheme is just rolling out and has plans and resources to scale-up to all of the country. The SMHP can piggyback on this scheme to facilitate scale-up.	1. Synchronizing the timing of teachers’ training on SMHP with the school academic calendar. *Rationale:* This will facilitate participation among teachers and school administrators, and also minimize the additional burden to teachers and administrators. 2. Clarifying roles among various individuals involved within the school system e.g. emphasizing the role of teachers to promote mental health (and not to provide targeted interventions) and to identify children requiring mental health care for prompt referral first to champion teachers who are expected to work with parents to guide the child’s access to the health system. *Rationale:* Teachers are already overextended on multitasks, and it was important to limit their role in the delivery of the programme to prevent overburdening them and to ensure their participation.	1.Inclusion of MOE’s counselling centres (which function as referral centres for school-aged children experiencing mental health problems within the MoE structure, providing counselling and psychological interventions). These counselling centres are then linked to Community Mental Health Centers (CMHC) which are community-based psychiatric health care facilities within the MoH structure for cases requiring specialized services. *Rationale:* Linking MoE and MoH services will foster co-operation at the primary care level, integration of mental health services for school children, and increased uptake of MoH specialized mental health services unavailable within the MoE system. 2. Exclusion of primary care physicians (PCPs) from the referral pathway of cases requiring specialized care. These children are referred directly by teachers from schools to the counselling centres and then to the CHMC, as the first point of contact in the health services delivery system. *Rationale:* PCPs in Iran may lack resources and expertise to provide adequate mental health care
Intervention adaptation^c^	1. Addition of a targeted intervention module on learning difficulties, self-harm and bullying which are prevalent mental health conditions in Egypt. *Rationale:* The addition of this module makes the SMHP programme more responsive to the specific mental health needs in Egypt.	1. Translation of the SMHP manual to Urdu, addition of a module on teacher self-care, use of culturally and age- appropriate case examples to illustrate key steps in interventions*.* *Rationale:* These changes will enhance the acceptability and easy application of the interventions. 2. Reframing the mental health conditions described in the SMHP programme as internalizing and externalizing problems. *Rationale:* This change will reduce labelling of children and stigma related to specific mental health diagnosis. 3. Adding a section on how to conduct parent–teacher interaction for teachers and others delivering the SMHP. *Rationale:* This change will improve teachers’ efficacy to interact more effectively with parents to facilitate the delivery of the programme.	1. Prioritizing teachers’ activities to focus on screening and identification of children with mental health needs (and not delivery of any targeted intervention). *Rationale*: This change will minimize any increased workload (or perception of increased workload) to teachers. 2. Prioritizing counsellors’ activities at MOE’s counselling centres to provide targeted interventions within the SMHP. *Rationale:* These trainings are viewed as additional qualification and knowledge expertise in mental health which is desirable to MOE counsellors. Thus, the trainings will provide incentives to counsellors under the MoE to support the delivery of the SMHP.

a Key strategies are approaches that determined the successful rollout of the entire programme, including those that were crucial for facilitating the described preconditions in each country.

b Contextual adaptations are changes to the internal environment (e.g. culture, norms and arrangements) within the implementing agencies, including key implementers or changes to the external environment (e.g. political, economic systems).

c Intervention adaptations are changes to the intervention (primarily the SMHP manual) to facilitate its delivery in a specific context.

## Discussion

The ToC and initial pathway for large-scale implementation of the SMHP in the EMR suggest that identifying national champions, formulating dedicated cross-sectoral (including the health and education sector) implementation teams, sustained policy advocacy and stakeholder engagement across multiple levels, and effective co-ordination among education and health systems especially at the local level are among the critical factors for large-scale implementation of the programme. While the role of champions and dedicated implementation teams have been previously recognized as crucial for facilitating large-scale implementation of any programme (Fixsen *et al.*, 2009; Higgins *et al.*, 2012; Alonge *et al.*, 2019), this paper extends findings to the success of large-scale implementation of population-based mental health programmes. Such teams are needed for driving organization- and system-level changes, facilitating high-level advocacy, responding to real-time implementation barriers and system bottlenecks and leveraging resources for ensuring the success of the programme (Alonge *et al.*, 2019). Furthermore, the teams enable a favourable implementation climate and leadership which are described as needed for the successful implementation of school mental health programmes in the implementation science literature (Greenhalgh *et al.* 2004; Fixsen *et al.*, 2005; Aarons *et al.*, 2009; Forman *et al.* 2009; Langley *et al.*, 2010).

Similarly, the role of cross-sectoral and multilevel stakeholder engagements has been previously described along pathways for supporting large-scale change of other programmes (The Health Foundation, 2012). This paper, however, highlights the constancy of this theory across different settings for large-scale implementation of SMHP. The stakeholder engagement in the three EMR countries was conducted purposively and employed a mix of group workshops and individual meetings. Conducting cross-sectoral engagement at both the national- and local-level was deemed critical for large-scale implementation of the SMHP by the core team of national implementers in all countries. This multilevel approach is necessary to account for local-level considerations pertinent to pilot implementation of the SMHP, whilst retaining a focus on large-scale implementation of the SMHP at the regional/national level which requires engagement with regional/national policy agendas and resource-allocation plans.

Cross-ministry engagement proved challenging in all settings, with the core team of national implementers in all countries working to establish links with counterparts in education ministries. However, these cross-ministry efforts sought to demonstrate to stakeholders an attitude of power-sharing and co-decision-making, and occurred at both the local-level relating to implementation (e.g. district representatives or NGO partners), and at the national level. Across the three countries, these cross-ministry links were founded upon informal relationships where common interests in working to meet the mental health needs of school children existed or could be established, and where there was a commitment to a collaborative cross-ministry approach to achieving this goal. Strategies that facilitated cross-ministry collaboration include location of the national implementation teams across the MoH and education, strategic power-sharing and decision-making between representatives of the ministries within the implementation team and in delivery of the SMHP, and ongoing negotiations between the ministries about how to frame and communicate the added value of the SMHP among the major priorities of both the health and education sectors.

The preconditions based on the initial implementation of the SMHP among the three countries highlight the distinction between the large-scale implementation of public health programmes and the scale-up of such programmes after an initial implementation research trial, and suggests that the preconditions may be emphasized differently for these similar, yet distinct objectives. Large-scale implementation requires deployment of a programme across multiple levels of the systems from the very start, and such commitment requires intense and rapid policy advocacy at both the national and local levels to facilitate real-time re-alignment of processes, re-allocation of resources and integration of programme activities into existing systems and structures. On the other hand, policy advocacy and integration of processes within the context of scaling-up after initial research may be more localized and gradual to align with research timelines. Whereas large-scale implementation of the SMHP comes with the added benefit of facilitating integration and institutionalization of programme activities, such large-scale effort may, however, expose the weaknesses and limited capacity of the existing systems to provide specialized and emergency mental health services to children referred from the SMHP. Such health system weaknesses and lack of access to specialized and emergency health services could derail the morale of parents and teachers, and significantly undermine the SMHP if not properly addressed. Thus, large-scale implementation of SMHP should be accompanied by strengthening aspects of the health system that are relevant for maintaining continuity of mental health care, with such concomitant strengthening of the relevant health system further requiring a greater degree of policy advocacy and action.

Some of the strategies deployed to facilitate the SMHP implementation draw from school-based mental health programmes (SMHPs) implemented in HICs (Wyn et al. 2000; Stein *et al.*, 2003; Bradshaw *et al.*, 2010; Li et al., 2011; Stallard *et al.* 2014; Guzman *et al.*, 2015; Humphrey *et al.*, 2016). The creation of a community-based primary health centre in Egypt recognizes the need to provide implementation support for the successful delivery of SMHPs as reported in the CBITS programme in the USA (Fixsen *et al.*, 2005; Langley *et al.*, 2010). The re-defining of school-based psychologists/counsellors’ role to provide targeted interventions in Egypt and Iran prioritizes intervention delivery by specialized cadre of school workers and address competing responsibilities of teachers, which are critical facilitators for the success of the FRIENDS programme in the UK (Stallard *et al.*, 2014). The synchronizing of the timing of teachers’ SMHP training with the school academic calendar in Pakistan acknowledges the need to align SMHPs to school’s policies and philosophy (Forman *et al.* 2009), and to address aspects of the organizational culture and climate relevant to successful implementation (Fixsen *et al.* 2005; Aarons *et al.*, 2009). Thus, findings suggest that key implementation principles and strategies for delivering SMHPs may be generalizable across HICs and LMICs settings.

The preconditions and pathway described for large-scale implementation of the SMHP in the EMR rely on the willingness of various relevant stakeholders to prioritize the mental health of school-aged children, the acceptability of a task-shifting approach for addressing gaps in mental health services, and the availability and motivation of school personnel (teachers, psychology graduates and school counsellors) to implement the SMHP. Hence, the large-scale implementation calls for an initial assessment of readiness of stakeholders to prioritize the mental health of school-aged children and deploy a task-shifting approach such as the SMHP, and understanding the incentives structure for key implementers prior to undertaking efforts to roll out the intervention (Alonge *et al.*, 2019).

The ToC approach has been previously applied as a tool to guide the evaluation of complex mental health interventions (De Silva *et al.*, 2014; Asher *et al.*, 2015; Chibanda et al., 2016; Breuer et al., 2018). This paper, however, extends the use of the ToC for developing and guiding real-time scale-up of mental health programmes. The methods used to develop this regional ToC and to identify the set of preconditions were highly iterative, moving between regional, national and local levels, and involved consultation with a wide range of actors working to promote school-based mental health, including representatives from the relevant ministries, NGO sector, schools and health systems. Ensuring this breadth of consultation is critical for the ToC to fully capture hypothesized pathways for the large-scale implementation of the SMHP (Breuer *et al.*, 2016); however, co-ordinating these consultations requires balancing trade-offs in arranging logistics. For instance, consultation with prominent actors such as ministers and their deputies may involve one-to-one meetings which preclude the opportunity for cross-learning and consensus building between stakeholders about the pathways to successful programme implementation that may present within a group setting. On the other hand, such individual meetings allowed for opportunistic feedback from a wider range of stakeholders than may otherwise have been involved.

Unlike other approaches for understanding complex interventions (Craig *et al.*, 2008; Mackenzie *et al.*, 2010), the ToC approach is flexible and can incorporate multiple perspectives in studying complex interventions and their implementation pathways; however, it can be resource-intensive and time-consuming (Breuer *et al*. 2018), as well as difficult to sustain the participation of a wide stakeholder group over an extended period.

Throughout the SMHP implementation, the CLG provides a forum for encouraging SHINE partner countries to continue engaging with their country-level ToC through ongoing review, revision, and updating as pilot SMHP implementation evolves. These country-level engagements embedded in a regional CLG allow local- and national-level factors to shape the development of the regional ToC and draw upon synergy between top-down (i.e. from regional level) and bottom-up approaches (from national and sub-national levels) to facilitate the implementation of a complex intervention like the SMHP. Key mechanisms for achieving country-level engagements are through scheduled programme meetings with key national stakeholders, as well as supervision structures between national trainers and SMHP implementers. The importance of an iterative process of review and revision is important to ensure that the benefits of the ToC—as a ‘live representation’ of the pathways to programme impact—are captured. To facilitate this iterative process, the core team of national implementers have become ToC champions who regularly drive re-engagement with the ToC as part of the process of SMHP implementation and evaluation, as has been found elsewhere (De Silva *et al.*, 2014). On the part of these implementers, the engagements have been driven by the benefits of seeing the applicability and relevance of the ToC to guiding SMHP implementation, allowing explicit articulation of implementation pathways that have facilitated the identification and resolution of potential challenges, as well as commitment of required resources.

The regional ToC for the SMHP identified through the methods described in this paper suggests preconditions and a pathway for improving the mental health outcomes of school-aged children in the EMR. The pathway operates through existing programmes of the MoH and MoE, and NGOs in the countries involved. For example, the pathway incorporates activities within a primary mental health centre supported by an NGO in Egypt, and counselling centres supported by MoH and MoE in Iran. These integration efforts will likely contribute to the sustainability of the SMHP in the long term (Kroenke and Unutzer, 2017). However, the key to sustaining the programme’s implementation activities rests with the team of national champions that include strategic policymakers and career public servants responsible for mental health services within each country. It is hoped that both the integration of the SMHP within programmatic activities under the MoH and MoE, and continued prioritization of the programme by career public servants in the respective ministries will facilitate the sustainability of the programme beyond the SHINE project period. The pathway described may have the unintended negative result of exacerbating weaknesses in national health systems within the EMR, and place an initial strain on existing mental health services due to the likely increase in referrals for specialized services, and the limited capacity to provide these services. However, the pathways may contribute to the unintended positive benefits of cross-sectoral collaboration between MoH and MoE that extend beyond improvements in mental health to also include improvements in nutrition, safety and prevention of other non-communicable health conditions for school-aged children.

The ToC approach as applied in this paper has some limitations. Firstly, as each team is led by MoH representatives there may be a bias in the ToC that prioritizes health over education-sector considerations across the ToC pathways. Whilst efforts have been made to counteract this bias through engagement with MoE stakeholders, the initial map was developed largely by MoH representatives; thus, some bias is likely to remain. Second, as the majority of country-level ToC validations occurred through individual meetings, the ToC represents the sum of these views rather than a consensus between stakeholders as would have emerged through workshops. This is a resource-intensive way of validating the ToC as it requires repeated explanation of the ToC and requires teams to effectively accumulate and integrate feedback into revised pathways, whilst ensuring these remain valid and not reflective of the potentially limited views of one stakeholder. However, as a regional ToC that aims to capture the commonalities of the ToC pathways across EMR countries, this limitation is considered acceptable as the ToC will always be abstracted from specific implementation considerations. Thirdly, as country teams moved forward with implementation of the SMHP in their respective countries, the focus of activities shifted to the local ToC, which prioritizes the pathways for local implementation of the SMHP, rather than large-scale SMHP implementation that the regional ToC sought to prioritize. This process acted to re-validate the commonalities of the regional ToC, whilst emphasizing the regional variations that arise as implementation becomes highly contextualized.

For next steps, this regional ToC will be incorporated into the WHO EMRO SMHP package to inform SMHP implementation across the EMR. It will act as an important starting point that countries can adapt according to their setting, and form the basis for developing monitoring and evaluation frameworks and conducting theory-based evaluation of the SMHP. Some research questions generated during the regional ToC process include testing assumptions around the acceptability of the SMHP with parents and other stakeholders as an approach for addressing mental health problems; exploring the efficacy of task-shifting to teachers to address mental health issues; and evaluating the effectiveness of various strategies included along the implementation pathway. These research questions and other evaluation questions are being actively explored as part of the next steps of the SHINE project. This regional ToC provides a useful platform for convening and engaging local stakeholders around the SMHP and facilitating local ownership of the programme, while also serving as a living document that provides an ongoing point of reference for revisiting as SMHP implementation pathways become further refined.

## Conclusion

This paper describes an approach to the development of a regional ToC that captures the common implementation pathways of a school-based mental health programme in the EMR. The paper further describes the key preconditions and strategies and programme/contextual adaptations that were used to facilitate those preconditions as implementation activities began in three countries within the region. The ToC and preconditions described in this paper are useful for facilitating effective implementation and integration of SMHP at scale, and will provide a theory-based framework for the evaluation of SMHP and similar programmes in EMR. This paper describes a novel application of ToC methods for programme development and guiding implementation processes in mental health and builds on existing literature in mental health where the ToC has been applied extensively as a tool to guide the evaluation of complex intervention.

## Supplementary Material

czaa124_Supplementary_Data

## References

[czaa124-B1] Aarons GA , Wells RS, Zagursky K, Fettes DL, Palinkas LA. 2009. Implementing evidence-based practice in community mental health agencies: a multiple stakeholder analysis. American Journal of Public Health 99: 2087–95. 19762654 10.2105/AJPH.2009.161711 PMC2759812

[czaa124-B2] Alonge O , Rodriguez DC, Brandes N et al 2019. How is implementation research applied to advance health in low-income and middle-income countries? BMJ Global Health 4: e001257. 10.1136/bmjgh-2018-001257 PMC6441291 30997169

[czaa124-B3] Asher L , Fekadu A, Hanlon C et al 2015. Development of a community-based rehabilitition intervention for people with schizophrenia in Ethiopia. PLoS One 10: e0143572. 26618915 10.1371/journal.pone.0143572 PMC4664267

[czaa124-B4] Attanayake V , Mckay R, Joffres M et al 2009. Prevalence of mental disorders among children exposed to war: a systematic review of 7,920 children. Medicine, Conflict, and Survival 25: 4–19. 10.1080/13623690802568913 19413154

[czaa124-B5] Benningfield MM , Stephan SH. 2015. Integrating mental health into schools to support student success. Child and Adolescent Psychiatric Clinics of North America 24: xv–xvii. 25773334 10.1016/j.chc.2014.12.005

[czaa124-B6] Bradshaw CP , Mitchell MM, Leaf PJ. 2010. Examining the effects of schoolwide positive behavioral intervetnions and supports on student outcomes: results from a randomized controlled effectiveness trial in elementary schools. Journal of Positive Behavior Interventions 12: 133–48.

[czaa124-B7] Breuer E , De Silva M, Lund C. 2018. Theory of change for complex mental health interventions: 10 lessons from the programme for improving mental healthcare. Global Mental Health 5: e24. 30128160 10.1017/gmh.2018.13 PMC6094401

[czaa124-B8] Breuer E , Lund C, Lee L, De Silva M. 2016. Using theory of change to design and evaluate public health interventions: a systematic review. Implementation Science 11: 63–79. 27153985 10.1186/s13012-016-0422-6 PMC4859947

[czaa124-B9] Charara R , Forouzanfar M, Naghavi M et al 2017. The burden of mental disorders in the eastern Mediterranean region, 1990-2013. PLoS One 12: e0169575. 28095477 10.1371/journal.pone.0169575 PMC5240956

[czaa124-B10] Chibanda D , Verhey R, Munetsi E, Cowan FM, Lund C. 2016. Using a theory driven approach to develop and evaluate a complex mental health intervention: the friendship bench project in Zimbabwe. International Journal of Mental Health Systems 10: 16–24. 26933448 10.1186/s13033-016-0050-1 PMC4772526

[czaa124-B11] ClinicalTrials.gov. 2019. *Identifier NCT04091633, School Health Implementation Network: Eastern Mediterranean (SHINE)*. Bethesda (MD): National Library of Medicine (US). https://clinicaltrials.gov/ct2/show/NCT04091633, accessed 16 September 2020.

[czaa124-B12] Collins PY , Patel V, Joestl SS et al 2011. Grand challenges in global mental health. Nature 475: 27–30. 21734685 10.1038/475027a PMC3173804

[czaa124-B13] Craig P , Dieppe P, Macintyre S et al 2008. Developing and evaluating complex interventions: the new Medical Research Council Guidance. BMJ 337: a1655. 18824488 10.1136/bmj.a1655 PMC2769032

[czaa124-B14] Damschroder LJ , Aron DC, Keith RE et al 2009. Fostering implementation of health services research findings into practice: a consolidated framework for advancing implementation science. Implementation Science 4: 50. 19664226 10.1186/1748-5908-4-50 PMC2736161

[czaa124-B15] Das JK , Salam RA, Lassi ZS et al 2016. Interventions for adolescent mental health: an overview of systematic reviews. Journal of Adolescent Health 59: S49–S60. 10.1016/j.jadohealth.2016.06.020 PMC5026677 27664596

[czaa124-B16] De Silva MJ , Breuer E, Lee L et al 2014. Theory of change: a theory-driven approach to enhance the Medical Research Council's framework for complex interventions. Trials 15: 267–78. 24996765 10.1186/1745-6215-15-267 PMC4227087

[czaa124-B17] Farooq S , Ayub M, Naeem F. 2015. Interventions following traumatic event in children and adolescents: an evidence-based response. Journal of Psychiatry 18: 3–5.

[czaa124-B18] Fixsen DL , Blase KA, Naoom SF, Wallace F. 2009. Core implementation components. Research on Social Work Practice 19: 531–40.

[czaa124-B19] Fixsen DL , Naoom SF, Blasé KA, Friedman RM, Wallace F. 2005. Implementation Research: A Synthesis of the Literature. Tampa, FL: Univrsity of South Florida, Louis de la Parte Florida Mental Health Institute, The National Implementation Resaerch Network. https://nirn.fpg.unc.edu/sites/nirn.fpg.unc.edu/files/resources/NIRN-MonographFull-01-2005.pdf, accessed 16 September 2020.

[czaa124-B20] Forman SG , Olin SS, Hoagwood KE, Crowe M, Saka N. 2009. Evidence-based interventions in schools: developers’ views on implementation barriers and facilitators. School Mental Health 1: 26–36.

[czaa124-B21] Gater R , Saeed K, World Health Organization, EMRO, Egypt 2015. Scaling up action for mental health in the Eastern Mediterranean Region: an overview. Eastern Mediterranean Health Journal 12: 535–45. 10.26719/2015.21.7.535 26442897

[czaa124-B22] Greenhalgh T , Robert G, Macfarlane F, Bate P, Kyriakidou O. 2004. Diffusion of innovations in service organisations: systematic review and recommendations. The Milbank Quarterly 82: 581–629. 15595944 10.1111/j.0887-378X.2004.00325.x PMC2690184

[czaa124-B23] Guzman J , Kessler RC, Squicciarini AM et al 2015. Evidence for the effectiveness of a national school-based mental health pro- gram in Chile. Journal of the American Academy of Child & Adolescent Psychiatry 54: 799–807. 26407489 10.1016/j.jaac.2015.07.005

[czaa124-B24] Higgins MC , Weiner J, Young L. 2012. Implementation teams: a new lever for organizational change. Journal of Organizational Behavior 33: 366–88.

[czaa124-B25] Imran N , Rahman A, Chaudhry N, Asif A. 2018. World Health Organization “School Mental Health Manual”-based training for school teachers in Urban Lahore, Pakistan: study protocol for a randomized controlled trial. Trials 19: 290–8. 29793553 10.1186/s13063-018-2679-3 PMC5968465

[czaa124-B26] Jordans MJD , Tol WA, Komproe IH, DE Jong JVTM. 2009. Systematic review of evidence and treatment approaches: psychosocial and mental health care for children in war. Child and Adolescent Mental Health 14: 2–14.

[czaa124-B27] Kieling C , Baker-Henningham H, Belfer M et al 2011. Child and adolescent mental health worldwide: evidence for action. The Lancet 378: 1515–25. 10.1016/S0140-6736(11)60827-1 22008427

[czaa124-B28] Kroenke K , Unutzer J. 2017. Closing the false divide: sustainable approaches to integrating mental health services into primary care. Journal of General Internal Medicine 32: 404–10. 28243873 10.1007/s11606-016-3967-9 PMC5377893

[czaa124-B29] Laal M , Ghodsi SM. 2012. Benefits of collaborating learning. Procedia - Social and Behavioral Sciences 31: 486–90.

[czaa124-B30] Langley AK , Nadeem E, Kataoka SH, Stein BD, Jaycox LH. 2010. Evidence-based mental health programs in schools: barriers and facilitators of successful implementation. School Mental Health 2: 105–13. 20694034 10.1007/s12310-010-9038-1 PMC2906726

[czaa124-B31] Li KK , Washburn I, DuBOIS DL et al 2011. Effects of the Positive Action programme on problem behaviours in elementary school students: a matched-pair randomised control trail in Chicago. Psychology & Health 26: 187–204. 21318929 10.1080/08870446.2011.531574

[czaa124-B32] Mackenzie M , O’Donnell C, Halliday E, Sridharan S, Platt S. 2010. Evaluating complex interventions: one size does not fit all. BMJ 340: 401–3. 10.1136/bmj.c185 20123834

[czaa124-B33] Merikangas KR , He JP, Burstein M et al 2010. Lifetime Prevalence of Mental Disorders in US Adolescents: Results from the National Comorbidity Study-Adolescent Supplement (NCS-A). *Journal of the American Academy of Child & Adolescent Psychiatry* **49**(10): 980–9. 10.1016/j.jaac.2010.05.017 PMC2946114 20855043

[czaa124-B34] Murphy JM , Abel MR, Hoover S, Jellinek M, Fazel M. 2017. Scope, scale, and dose of the world’s largest school-based mental health programs. Harvard Review of Psychiatry 25: 128–228. 10.1097/HRP.0000000000000149 28787304

[czaa124-B35] Murray LK , Dorsey S, Bolton P et al 2011. Building capacity in mental health interventions in low resource countries: an apprenticeship model for training local providers. International Journal of Mental Health Systems 5: 30–42. 22099582 10.1186/1752-4458-5-30 PMC3284435

[czaa124-B36] Patel V , Saxena S, Lund C et al 2018. The Lancet Commission on global mental health and sustainable development. The Lancet 392: 1553–98. 10.1016/S0140-6736(18)31612-X 30314863

[czaa124-B37] Peters DH , Al-Saharty S, Siadat B, Janovsky K, Vujicic M. 2009. Improving Health Service Delivery in Developing Countries: From Evidence to Action. Washington, DC. https://openknowledge.worldbank.org/bitstream/handle/10986/12334/48790.pdf, accessed 16 September 2020.

[czaa124-B38] Purgato M , Gross AL, Betancourt T et al 2018. Focused psychosocial interventions for children in low-resource humanitarian settings: a systematic review and individual participant data meta-analysis. Lancet Global Health 6: e390–e400. 29530422 10.1016/S2214-109X(18)30046-9

[czaa124-B39] Rahman A , Hamoda HM, Rahimi-Movaghar A, Khan M, Saeed K. 2019. Mental health services for youth in the eastern Mediterranean region: challenges and opportunities. Eastern Mediterranean Health Journal 25: 80–1. 30942470 10.26719/2019.25.2.80

[czaa124-B40] Reed RV , Fazel M, Jones L, Panter-Brick C, Stein A. 2012. Mental health of displaced and refugee children resettled in low-income and middle-income countries: risk and protective factors. The Lancet 379: 250–65. 10.1016/S0140-6736(11)60050-0 21835460

[czaa124-B41] Saxena S , Funk MK, Chisholm D. 2015. Comprehensive mental health action plan 2013-2020. Eastern Mediterranean Health Journal 12: 461–3. 26442884

[czaa124-B42] Stallard P , Skryabina E, Taylor G et al 2014. Classroom-based cognitive behaviour therapy (FRIENDS): a cluster randomised controlled trial to Prevent Anxiety in Children through Education in Schools (PACES). The Lancet Psychiatry 1: 185–92. 26360730 10.1016/S2215-0366(14)70244-5

[czaa124-B43] Stein BD , Jaycox LH, Kataoka SH et al 2003. A mental health intervention for school children exposed to violence: a randomized controlled trial. JAMA 290: 603–11. 12902363 10.1001/jama.290.5.603

[czaa124-B44] The Health Foundation. 2012. Evidence Scan: Cross Sector Working to Support Large-Scale Change. London, UK. https://www.health.org.uk/sites/default/files/CrossSectorWorkingToSupportLargeScaleChange.pdf, accessed 16 September 2020.

[czaa124-B45] Vogel I. 2012. Review of the Use of ‘Theory of Change’ in International Development. London, UK. http://www.theoryofchange.org/pdf/DFID_ToC_Review_VogelV7.pdf, accessed 16 September 2020.

[czaa124-B46] World Health Organisation. 2011. Maternal, Child and Adolescent Mental Health: Challenges and Strategic Directions for the Eastern Mediterranean Region. Cairo: World Health Organisation Office for the Eastern Mediterranean Region. https://apps.who.int/iris/bitstream/handle/10665/116689/dsa1214.pdf? sequence=1&isAllowed=y, accessed 16 September 2020.

[czaa124-B47] World Health Organisation. 2018. *Mental Health Atlas.(Licence: CC BY-NC-SA 3.0 IGO)*. Geneva. https://apps.who.int/iris/bitstream/handle/10665/272735/9789241514019-eng.pdf? ua=1, accessed 16 September 2020.

[czaa124-B48] World Health Organisation Office for the Eastern Mediterranean Region. 2014. Manual of School Mental Health. World Health Organisation Office for the Eastern Mediterranean Region. Cairo, Egypt.

[czaa124-B49] Wyn J , Cahill H, Holdsworth RW, Rowling L, Carson S. 2000. MindMatters, a whole-school approach promoting mental health and wellbeing. Australian & New Zealand Journal of Psychiatry 34: 594–601. 10.1080/j.1440-1614.2000.00748.x 10954390

