# Supplementary material for: Identifying pathways for large-scale implementation of a school-based mental health programme in the Eastern Mediterranean Region: a theory-driven approach
Source: Health Policy Plan. 2020 Nov 6;35(Suppl 2):ii112–23. doi: 10.1093/heapol/czaa124 (PMC7646738; doi:10.1093/heapol/czaa124)
Supplement: czaa124_Supplementary_Data [file czaa124_supplementary_data.zip › czaa124-suppl_data/Supplementary File 1.pdf]

This appendix provides an overview of the EMRO SMHI. The excerpts provided are examples drawn from the detailed manual, which is over 80 pages.

MANUAL OF SCHOOL MENTAL HEALTH

World Health Organization
Eastern Mediterranean Regional Office

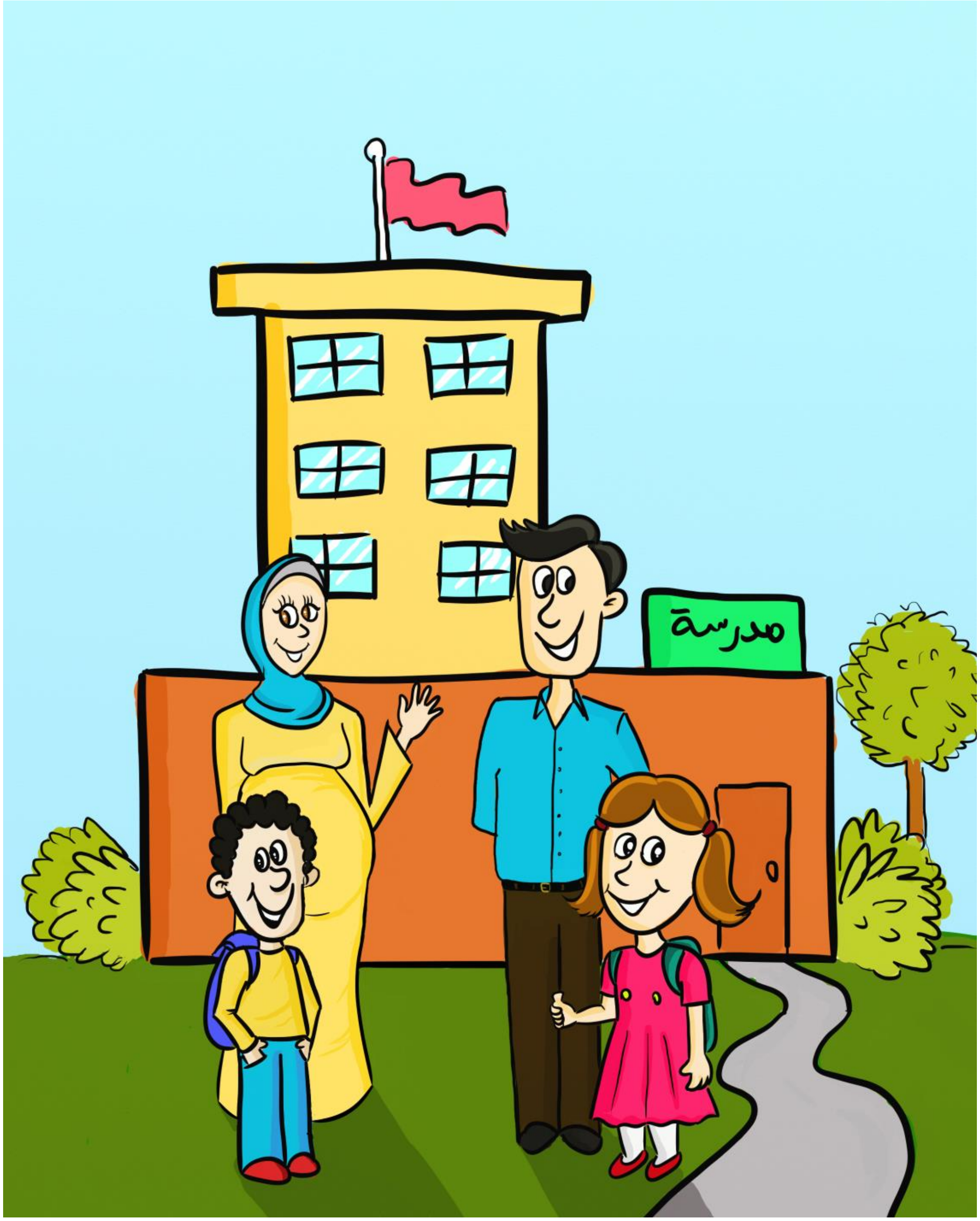

December 2014

Overview of contents

Module 1. Social-Emotional Childhood Development

1.1 Developmental Tasks of Preschoolers
1.2 Developmental Tasks of primary-school-age children (6-12 years):
1.3 Developmental Tasks of secondary-school-age children (12-18 years):
1.4 Moral Development
1.5 Brain Development and Implications for Schooling

Module 2. Mental Health Promoting Schools (Promotion and Prevention)

2.1 Core Values of a Mental Health Promoting School
- **2.1.1** The Role of Parents in their Children's Education

2.2 General Behavioral Management Strategies for Schools
- **2.2.1** Discipline and Management of Disruptive Behavior
- **2.2.2** Counseling
- **2.2.3** Circle Time

2.3 Life Skills Training
2.4 Physical Health Promoting Efforts that Impact Mental Health
- **2.4.1** Nutrition
- **2.4.2** Vision/ Hearing/ Speech
- **2.4.3** Physical exercise

2.5 Media and Mental Health
- **2.5.1** Screen Time
- **2.5.2** Internet Addiction
- **2.5.3** Cyberbullying

2.6 Suicide Prevention

Module 3. Addressing Student Mental Health Problems in Your Classroom (and when to refer)

3.1 Recognizing Warning signs for Different Mental Health Problems:
- **3.1.1** The excessively sad or emotionally withdrawn child
- **3.1.2** The overly anxious child
- **3.1.3** The child who has difficulties socializing
- **3.1.4** The child who has attentional difficulties
- **3.1.5** The child who has suddenly started thinking or behaving oddly
- **3.1.6** The child who has difficulty learning specific subjects
- **3.1.7** The child who is slower than other children of his or her age
- **3.1.8** The child with severe behavior issues
- **3.1.9** The child who might be taking drugs or other substances

3.2 Matching interventions to Symptoms
- 3.2.1 Improving Attention, Organization, Hyperactivity, Impulsivity
- 3.2.2 Responding to Traumatic, Disaster, or Very Stressful Situations
- 3.2.3 Improving Social Communication, Engagement with Others
- 3.2.4 Improving Behavior, Cooperation, Collaboration, and Empathy
- 3.2.5 Decreasing Anxiety and Stress
- 3.2.6 Improving Mood and Mood Regulation
- 3.2.7 Addressing Unusual Thoughts

3.2 When to Refer to a Specialist for Evaluation and Treatment?
3.3 Roles and Responsibilities within the School in Regards to Mental Health

Module 4. Case studies

4.1 ADHD
4.2 Children affected by Traumatic, Disaster, or Very Stressful Situations
4.3 Autism

4.4 Conduct Disorders
4.5 Anxiety
4.6 Depression
4.7 Separation Anxiety
4.8 Psychosis
4.9 Bullying
4.10 Suicide
4.11 Substance Abuse

Resources

- ❑ **Guide for School Health Programme Managers for implementation of the Promotion of Emotional and Psychosocial Well-being in Schools**
- ❑ **Examples of School Intervention Programs from the Eastern Mediterranean Region**
- ❑ **Screening Tools that can be used at Schools**

Target Audience:

This manual is primarily intended for those involved in the educational process including teachers, school administrators, nurses, social workers, school counselors, in addition to educational policy makers and non-governmental organizations.

This manual can be used together with supporting tools that are part of this package including slides and handouts. Additional resources are also available in the Appendix.

Objectives of this Manual:

- Helping educators understand the importance of mental health in a school setting.
- Enhancing educators' understanding about child development.
- Incorporating mental health into healthy schools initiatives.
- Providing age – appropriate behavioral management strategies including disciplining and management of disruptive behaviors.
- Understanding how mental health can be promoted at school settings.
- Identifying the warning signs of mental illness in school children and distinguishing that from emotional distress.
- Providing appropriate interventions for a variety of psychiatric disorders.
- Providing further resources that can be accessed by educators.

Guide to Using this Manual:

This manual is intended to be a guide for educators to better support the mental health needs of their students and to take practical steps implementable in school settings. We emphasize interventions and supports that can be implemented at relatively low cost. The manual is divided into modules that can each be used separately. It is intended to be a concise and practical guide.

The manual uses visual aids to assist the reader. While reading the manual

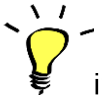

indicates "actionable items" including practical tips or interventions to be implemented.

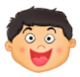

More applicable to children of young age <8 years

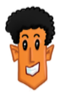

More applicable to adolescents >13 years

Module 3
Addressing Student Mental Health Problems in Your Classroom (and when to refer for additional help)

Childhood and adolescence are periods characterized by rapid developments not only in the physical domain, but also emotionally and cognitively. Most people go through this period without significant mental health problems however, almost everyone experiences some emotional distress during their journey towards adulthood, which they learn to overcome and adapt to.

It is not necessary for teachers to determine whether a child has a mental disorder, nor to diagnose a child with a mental disorder. Attempting to diagnose children risks inappropriately labeling a child, alienating children and their parents, and requires specialized training.

Rather, it is important for teachers to understand how to support the mental health of all students in the classroom, including those with mental health problems and disorders, and to determine when mental health problems are severe enough to require additional help from family members and/or a mental health specialist.

3.1 Behavioral Manifestations of Common Mental Health Problems

In the following section we will present the behavioral manifestations of common mental health problems that could be encountered in a school setting.

*Students with **Anxiety Problems** may:*

- ❑ Feel afraid, anxious, angry, irritable and/or frustrated
- ❑ Cry excessively, tantrum.
- ❑ "Freeze" or be unable to participate in activities.
- ❑ Demonstrated clinginess with caregivers and teachers.
- ❑ Fidget.
- ❑ Be easily frustrated.
- ❑ Worry so much about getting everything right that they take much longer to finish their work.
- ❑ Refuse to begin out of fear that they won't be able to do anything right.
- ❑ Avoid school out of fear of becoming embarrassed, humiliated, or failing.
- ❑ Be behind in their work due to numerous absences.
- ❑

Students with **Post-Trauma Problems** *may:*

- ❑ Feel anxious or irritable
- ❑ Have drastic mood changes or appear unusually sad
- ❑ Act younger than age
- ❑ Be clingy and/or whiny
- ❑ Be impulsive, and/or aggressive.
- ❑ Be unable to perform previously acquired skills, even basic functions like speech.
- ❑ Have difficulty concentrating
- ❑ Be preoccupied, and become easily confused
- ❑ Lose interest in activities
- ❑ Become quiet and/or sad, and avoid interaction with other children.
- ❑ Not show feelings or appear "numb"
- ❑ Avoid activities or places related to trauma
- ❑ Repetitive play with themes related to trauma
- ❑ Nightmares/flashbacks
- ❑ Exaggerated startle response
- ❑ Difficulty sleeping

Students with ***Depression or Sadness Problems*** *may:*

- ❑ Cry easily, look sad, feel alone or isolated
- ❑ Appear anxious or afraid
- ❑ Act angry or irritable
- ❑ Demonstrate marked changes in school behaviors.
- ❑ Find it harder to stay on task. Lose concentration.
- ❑ Have frequent absences from school.
- ❑ Experience change in academic performance.
- ❑ Lose of motivation.
- ❑ Abandon favorite hobbies or sports, decreased interest in being with peers, withdrawn.
- ❑ Change eating and sleeping habits. Have changes in appetite or weight.
- ❑ Have changes in feeling, thinking and perceiving.
- ❑ Express inappropriate guilt.
- ❑ Express feelings of not being good enough, worthlessness, failure.
- ❑ Express hopelessness: nothing to look forward to.
- ❑ Speak in a monotonous or monosyllabic manner.
- ❑ Be irritable; e.g., snapping at people for no apparent reason.
- ❑ Be restless or slowed down.
- ❑ Misuse drugs and alcohol.
- ❑ Eats/sleeps too much or too little
- ❑ Students with depression are at increased risk for self-injury, suicidal thoughts and attempts.
- ❑ Educators should be wary of any comments about or signs of self-injury or suicide.
- ❑ Every comment should be taken seriously and brought to the immediate attention of the child's parent, mental health specialist, and/or school nurse.

Students with ***Mood Problems*** *may:*

- ❑ Show fluctuations in mood, energy, and motivation. These fluctuations may occur hourly, daily, in specific cycles, or seasonally.
- ❑ Alternate between fearfulness and recklessness
- ❑ Appear angry, irritable and/or frustrated
- ❑ Have episodes of overwhelming emotion such as sadness, embarrassment, elation or rage.
- ❑ Have difficulty concentrating and remembering assignments, understanding assignments with complex directions, or reading and comprehending long, written passages of text.
- ❑ Demonstrate poor social skills and have difficulty getting along with peers.
- ❑ Younger children:
  - ❑ frequently exhibit fast mood swings, many times within a day.
  - ❑ Younger children are more likely to be irritable and prone to destructive tantrums than to be overly happy and elated.
- ❑ Older adolescents:
  - ❑ Show similar patterns of mood instability as adults with changes from high to lows, involving high intensity of mood.

Students with ***Hyperactivity and Impulsivity Problems*** *may:*

- ❑ Have difficulty paying attention or staying on task
- ❑ Not complete tasks and making careless errors/mistakes
- ❑ Make choices without thinking them through
- ❑ Blurt out answers before teacher finishes question or calls on them
- ❑ Interrupt teacher and other students
- ❑ Talk too loudly
- ❑ Fidget/Have difficulty remaining still and staying in seat
- ❑ Other children may get easily frustrated with them and they may become frustrated with peers and themselves

Students with ***Inattention Problems*** *may:*

- ❑ Not listen when spoken to
- ❑ Have difficulty paying attention or staying on task
- ❑ Not complete tasks and making careless errors/mistakes
- ❑ Forget tasks and materials (jackets, books, pencils, homework)
- ❑ Daydream or appear “spacey”
- ❑ Have a very messy/disorganized desk area
- ❑ Lose objects
- ❑ Avoid activities that require sustained mental effort

*Students with **Oppositional Problems** may:*

- ❑ Challenge class rules.
- ❑ Refuse to do assignments.
- ❑ Lose temper
- ❑ Argue or fight with other students.
- ❑ Argue with the teacher.
- ❑ Deliberately try to provoke people
- ❑ Disobey rules and directions.
- ❑ Intentionally create conflict with peers.
- ❑ Blame others for their actions and behaviors.
- ❑ Interpret motives and behaviors of others in a negative light.
- ❑ Seek revenge for perceived wrongs.

*Students with **Conduct Problems** may:*

- ❑ Engage in power struggles
- ❑ React badly to direct demands or statements such as: “you need to…” or “You must…”
- ❑ Consistently challenge class rules
- ❑ Refuse to do assignments
- ❑ Argue or fight with other students
- ❑ Create disruptions in the class
- ❑ Blame others and do not take responsibility for their behavior
- ❑ Steal from others
- ❑ Destroy property in the classroom
- ❑ Disrespect the adults and other students
- ❑ Endanger the safety and wellbeing of others

*Students with **Substance Use Problems** may:*

- ❑ Be moody and irritable, including sudden mood or personality changes
- ❑ Have low self-esteem and depression
- ❑ Behave irresponsibly
- ❑ Withdraw socially
- ❑ Pull away from family, teachers, other trusted adults
- ❑ Be argumentative and disruptive
- ❑ Break rules
- ❑ Decline in academic performance
- ❑ Have memory and learning problems
- ❑ Demonstrate poor judgment in situations
- ❑ Be late and absent from school
- ❑ Have problems with family and peer relationships, and a lack of empathy for others.
- ❑ Engage in other risky activities
- ❑ Change former activities or friends
- ❑ Demonstrate general lack of interest

*Students with **Psychosis Problems** may:*

- ❑ Have perceptions (visual, auditory, tactile) in the absence of external stimuli.
- ❑ Have thoughts or beliefs that are unusual and not shared in the individual’s culture.
- ❑ Speak in a way that is difficult to follow.
- ❑ Behave unpredictably (e.g., childish silliness, agitation, complete lack of motor or verbal activity).
- ❑ Feel like others want to hurt him/her or are plotting against them.
- ❑ Withdraw from peers in the classroom.
- ❑ Act unmotivated to participate in class and to complete homework.

3.2 Strategies to Address Behavioral Manifestations of Common Mental Health Problems

Below we describe some behaviors that may manifest in the classroom, and specific strategies to address those behaviors. We indicate whether the strategy is appropriate for implementation by teachers and/or parents and/or peers. Strategies are organized into three categories:

Tier 1 strategies – To address mild problems. Strategies are simple to implement and will likely benefit all students in the classroom.

Tier 2 strategies – To address moderate problems, or if Tier 2 interventions are not sufficient to address problems. Strategies require specific activities tailored to the child with problems.

Tier 3 strategies – To address severe problems, or if Tier 2 interventions are not sufficient to address problems. Strategies require specific activities tailored to the child with problems, and may necessitate the involvement of additional teaching staff.

Matching Interventions to Symptoms:

	**3.2.1 Improving Attention, Organization, Hyperactivity, Impulsivity**	
**Troubling Behaviors You Might See In the Classroom**	**Intervention**	**Group T=Teacher P=Parent F=Friends**
**Inattention**	Preferential Seating Sit at front of class	T, P
	Post Rules, the Daily Routine, and a School Calendar at a regular place and go over daily (e.g., front of classroom, at home)	T,P
	Break steps down and have child repeat back	T, P
	Tape the steps to solve a problem on the student's desk	T,P
	Allow extra time to complete tasks, or for the student to complete tasks at designated times (not at recess, as the student often needs to discharge energy)	
	Provide the student a copy of notes or allow to tape-record	T
	Allow the student to do tests verbally (instead of writing)	T,P
	the child starts by completing sentences already started, or paragraphs structured for them ("I am in favor of ____. The first reason supporting this is _____. An example that shows this is_____. Another example is _____. The second reason is _____. etc."	T,P
	Underline, circle, or highlight key terms on reading material for the student	T,P
	Have a staff member help the student write responses and model how to stay on task	T,P
**Organization**	Have students check in after doing the first 1-2 problems to ensure they are following the correct steps	T,P
	Praise/Reinforce the child for doing the "right thing" when follows steps, organizes desk, and other goals prioritized for the student	
	Keep extra materials (pencils, books when possible) at school and at home	T,P
	Help the child clean out desk and to organize papers in colored folders	T,P
	Have the child use a daily assignment book and check it before the student leaves for home	T,P
	Identify a "coach" staff person for the student to meet with at the end of each day to prepare materials before going home	T,P,F
**Hyperactivity**	Provide breaks for the student to move about	T,P
	Break tasks down into 10-20 min segments so that the students can move/shift within the classroom appropriately	T,P
	Provide alternative responses for physical activity; have the child raise a hand, count to 5, then raise the other hand; have the student wiggle fingers/toes to relax (and not be disruptive)	T,P, F
	Provide goal-directed tasks such as moving papers or taking attendance to office	T,P,F
**Impulsivity**	Clarify rules of personal space (stand 1 floor tile/3 arm lengths apart, use your inside voice after other person has stopped speaking, etc.)	T,P,F
	Allow the child to have a designated place in lines with children (between 2 effective peers)	T,P,F
	Allow the student to leave early with another staff or peer to the next place/class	
Blurting Out	Have the child practice listening, waiting, saying back what person said, and then respond	T,P,F

	3.2.2 Responding to Traumatic, Disaster, or Very Stressful Situations	
**Troubling Behaviors You Might See In the Classroom**	**Intervention**	**Group T=Teacher P=Parent F=Friends**
Distress, crying, irritability	Make the school a safe, predictable place with normal routines (however, academic demands may need to be decreased for days to weeks depending on the severity of the trauma	T,P
	Allow the students to deal with traumatic reminders at their own pace (avoid having a memorial in a prominent place in the school and instead position a book in a school room where the students can write their thoughts or comments)	T,P,F
	Consider school events to minimize trauma (avoid unnecessary fire drills or discussion of historical events that force the students to recall the trauma)	T,P,F
	Teach relaxation techniques to diminish escalating distress; teach students to tighten and loosen their fingers, toes, etc.; teach students to deep breathe in slowly through their nose, hold as they count to 5, and slowly exhale through their mouths	T,P,F
	If students shows distress, help them identify what triggered their distress (do this outside of class, and consider alternatives [e.g., reading different material, doing something to distract themselves while in class)	T,P,F
	Identify with the student "signals" such as raising a finger to allow the student to be excused if distressed	T,P,F
	Identify coping skills such as alternative activities that the student can do such as independent projects or for outside class	T,P,F
	Help students recognize and think through their options and likely consequences when something distresses or reminds them	T,P,F
	Review with the parents or other teachers the "story" of a student's trauma so that one simple, acceptable "version" circulates	T,P
	Allow the student to write reactions in a journal that can be put away or reviewed with the teacher or other staff later	T,P
	Allow the student to go see other staff to regroup and then return to class	T,P
Intrusive Thoughts or Flashbacks	Help the child recognize that their current situation is safe, so they are grounded and not afraid in the classroom	T,P,F
	Provide "a minute" for the student to think of other things or do something else (get a drink of water, do a different task, move to another place in the room)	T,P
	Encourage the student to identify friends who help support them and protect them in that moment	T,P,F
	Allow the child to write down an intrusive thought or flashback to discuss with other staff	T,P

	3.2.3 Improving Social Communication, Engagement with Others	
**Troubling Behaviors You Might See In the Classroom**	**Intervention**	**Group T=Teacher P=Parent F=Friends**
Social Rules	Describe rules in positive language for students (Respect for others, walk in a line, speak after the other person finishes, keep your hands to yourself, etc.)	T,P,F
	Point out in stories, movies, television shows, etc., how people stand, look at each other, and start, continue, and stop conversations appropriately	T,P,F
	Practice having students listen to another student, and to ask 1-2 questions rather than change	T,P,F

	the topic or talk about themselves; this is sometimes easier when students identify particular interests and can be matched up	
	Bend down to the student's eye level rather than request student look at teacher	T,P
	Have students predict what will happen before continuing to read a story or describe historical events	T,P
	Wait 3 seconds after asking the student a question for the student to process and answer	T,P
	Use language clear to these students (simple, concrete instead of sarcasm, metaphors, idioms)	T,P,F
	Explain figurative language and alternative "meanings" of statements, particularly in stories, poems	T,P
	Explain nonverbal communications (facial expressions for happiness, anger, disgust, surprise, etc.) to help students accurately recognize emotions of others	T,P,F
	When students get excited or escalate, slow down, speak softer, be calmer	T,P
	Tape-record or videotape the student and review the student's social behavior (individually, outside of class)	T,P
	Identify peers the student can work, play, and eat snacks/meals with	T,P
	Provide signals and time for students to transition	T,P
	Provide visuals (e.g., path to the playground) to point to rather than only verbal directions	T,P
	Substitute acceptable behaviors for unacceptable ones (touching a piece of fabric instead of pants, squeezing a soft ball instead of flipping or waving a pen, etc.)	T,P
	Reward the student with desired tasks, activities, when does the right thing (e.g., computer time, desired book, play with preferred peer, etc.)	T,P
	Teach alternative activities if the student does "unusual activities" (e.g., lines up cars, show how to roll them)	T,P,F
	Provide Velcro choices of activities the student can change as they decide some of their routine and transitions	T,P
	Provide a "social story" of events so the student thinks through all the steps and will not become upset if surprises/deviations arise (www.thegraycenter.org)	T,P
	Position the student in a social skills group at lunch or other times to practice asking questions and speaking conversationally	T,P,F
	When available, have other staff familiar with sensory devices (e.g., occupational therapy) help the student identify alternative sensory experiences to calm down (deep joint compression, weighted blankets/clothes, headphones to block out noise, etc.), and identify school tasks (lifting, being in quiet places) that enhance learning	T,P

	**3.2.4 Improving Behavior, Cooperation, Collaboration, and Empathy**	
**Troubling Behaviors You Might See In the Classroom**	**Intervention**	**Group T=Teacher P=Parent F=Friends**
Refusal	Provide the student a few appropriate choices ("you can do this work during lunch, or I'll help you now do the first problem")	T,P
	Use "I need you" rather than "you need to" statements	T,P
	Use consistent cues, words, and signals to identify inappropriate behaviors; state what you want instead of what you do not want; model politeness ("please walk down the hall on the right side seeing if you can be the quietest you've ever been")	T,P,F
	Slowly think through the student's alternatives and likely consequences when refusal occurs; allow the student to consider and choose options	T,P

	Acknowledge the student's frustration or disappointment when something doesn't go as they want, and then invite the student to figure out another solution for now	T,P
	Identify the student's good efforts even if the results are not successful	T,P
	Focus on fixing problems rather than who is to blame; reward collaborative efforts between the student and others	T,P,F
	Have students describe how they think others feel when a conflict occurs	T,P,F
	Have students role-play how to resolve conflicts	T,P
	Confront lies/distortions outside of class	T,P
	Allow the student to correct mistakes or misdeeds	T,P
	Identify a "time-out" space in the classroom where the student can go to calm down	T,P
	Minimize escalations by speaking softly, sparsely, and demonstrating patience as allow the student to do the right things	T,P
	Identify a staff member to walk or talk with the angry student to process the event outside of class	T,P
	With parents, identify prosocial events/activities or other helpful peers and students for the student to spend more time with	T,P,F

	**3.2.5 Decreasing Anxiety and Stress**	
**Troubling Behaviors You Might See In the Classroom**	**Intervention**	**Group T=Teacher P=Parent F=Friends**
Escalating Anxiety	Speak slowly and calmly, encourage breathing slowly;	T,P
	"Break tasks down (right now we just need to walk to the water cooler to get a drink")	T,P
	Identify stressors and alternatives (if student is afraid to speak in front of the class, allow them to speak into a tape recorder or perform at lunch in front of 1-2 familiar peers)	T, P
	Help the students consider the probability of events ("I'm afraid to get on the bus because it will crash." "Hmmm…what's the chances it will crash? How many buses do you see driving out there that are not hitting other cars.")	T,P
	Allow extra time to complete tasks, or for the student to complete tasks at designated times (not at recess)	T,P
	Help students evaluate all the evidence for their conclusions ("I'm no good at math." "Hmmm….what have your math grades been for the past week? All good except today? Wonder why you had one hard day and the others all went well?")	T,P
	Model and practice positive self-talk ("I can do this." "Even though I missed the last problem, I can get the next one correct."	T,P,F
	Have students use a fear thermometer to identify what most frightens them and what to do when they are at different levels	T,P
	Allow the student to do tests informally with the teacher (instead of writing, speaking in front of peers)	T,P
	Use successive approximations: if the child is afraid of dogs, allow the child to see pictures of a dog, see videos of dogs, then see others play with dogs outside, then touch a dog on a leash or separated by a fence, etc.)	T,P
	Have the student meet with a counselor to express sources of anxiety and how to manage distress, or identify appropriate behaviors if they are engaging in compulsive rituals	T,P

	Identify a staff response to anxious students so that others act in a predictable fashion (instead of increasing anxiety by doing unusual things to scare the child into compliance)	T,P
Refusal to Separate from Parents to Attend School	Make school more magnetic (something to look forward to on arriving, such as playing with peer, feeling animals, etc.), and home less magnetic (parents need not be mean but just not play or show attention to the child for staying at home)	T,P
	Encourage parents to make home more boring if the student resists going to school (no sleeping in, watching television or playing video games, etc.)	T,P
	Allow parents to send notes in the student's lunch (rather than phone the student while at school if they have trouble separating)	T,P
	Have the student use "strength" cards (e.g., Pokemon, superheroes, etc.) to recall strengths and powers to manage stress	T,P
	Allow the child to spend time at first in the library or with other staff to ease into the building (and reward efforts to get to the classroom)	T,P
	Take pictures of the student doing well in the classroom or in school activities and provide those for the student to view and take home	T
	Introduce the student to next year's teacher and to have parents visit next year's classroom during a vacation interval	T,P
	Identify a hierarchy of staff to meet the child on arrival to school, and other staff where the child can go if distressed during class time	T
	Have the school counselor help the child practice the transition to and from school to develop a routine that is easier	T

	**3.2.6 Improving Mood and Mood Regulation**	
**Troubling Behaviors You Might See In the Classroom**	**Intervention**	**Group T=Teacher P=Parent F=Friends**
Negative Mood	Check in with the student to quantify his mood (on a 10 point scale with 10 being happy), and steps the student can take if mood is low (take a walk, listen to music, exercise, seek out a positive peer)	T,P,F
	Connect academic assignments with student interests, or allow special projects for the student to remain interested/engaged	T,P
	Identify activities or class projects where the student can work with supportive peers	T, P
	Help the student evaluate "all the evidence" surrounding negative thoughts	T,P
	Identify desired activities during the school day for the student to "look forward to" each day	T,P,F
	Help students evaluate all the evidence for their conclusions ("I'm no good at math." "Hmmm….what have your math grades been for the past week? All good except today? Wonder why you had one hard day and the others all went well?")	T,P
	Model and practice positive self-talk ("I can do this." "Even though I missed the last problem, I can get the next one correct."	T,P,F
	Allow the student to do alternative tasks or to be in other parts of the room if weepy or sad	T,P
	Have the student start with familiar, previously successful tasks to get going and then move to new and/or more challenging tasks	T,P
	Grade the student based on work completed rather than work assigned	T,P
	Provide class notes to the student	T,P
	Identify study partners who can support and assist with assignments	T,P,F

	Have the student write in a journal about moods, and write songs, poems	T,P
	Allow the student to do tests informally with the teacher (instead of writing, speaking in front of peers)	T,P
	Outside of class, help the student identify evidence contributing to distressing mood states	T,P
	Have the student meet with a counselor who can challenge negative thoughts or focus on the relationships between thoughts, feelings, and actions	T,P
	Create a support group for appropriate students	T,P,F
	Identify a staff response to self-injurious behaviors so that students are assessed without being stigmatized	T,P

	**3.2.7 Addressing Unusual Thoughts**	
**Troubling Behaviors You Might See In the Classroom**	**Intervention**	**Group T=Teacher P=Parent F=Friends**
Distorted Thoughts or Comments	Instead of arguing about distorted thoughts, shift to academics ("Okay, I heard your comments, and now we'll move forward with math.")	T,P
	Provide grounding comments to help the students share the reality of others ("Well, despite your fears about others, you are in your 5th grade class with all your usual classmates.")	T,P
	Emphasize a simple structure and routine that remains familiar, and can be shifted if necessary if a particular topic or task is triggering the student	T, P
	Rely on concrete, observable activities (e.g., math instead of reading a complex character novel) when the student reports more unusual thoughts	T,P
	Employ a series of steps to deescalate students when unusual thoughts increase: (1) change topic, (2) change activity, (3) change setting (room or place, (4) change staff (have the student engage with different staff)	T,P
	Allow extra time to complete tasks, or for the student to complete tasks at designated times (not at recess)	T,P
	Help students evaluate the evidence for their conclusions ("You think that student wants to hurt you—has he attempted to hurt anyone or you ever before?")	T,P
	Model and practice positive self-talk ("I will get through this—I made it through yesterday and will get through this today, too." "Even though I hear someone's voice, it is okay and will not hurt me."	T,P
	Communicate with parents, treaters to clarify ahead of time what might be difficult topics and what might be good topics/activities for this student	T,P
	Clarify if certain activities (e.g., playing music or sports) help distract the student from unusual thoughts	T,P
	Have the student meet with a counselor to discuss bizarre or frightening thoughts	T,P
	Identify a hierarchy of places and staff the student can access if the student cannot remain in the room, and how the student can transition safely between staff	T,P

Example case study

1. Case study (ADHD/disruptive behaviors):

Hassan is a 7-year-old boy in 2nd grade. His teacher notices that he is not able to sit still in class. He is always disrupting class and disturbing other students. When his teacher calls on him to stop he says he can’t help it as “my body is always on the go”. Other kids complain that he is always in their space. He frequently finds himself distracted by other students and everything that happens in the class seems to capture his attention. His teacher feels that he is behind on his learning despite that he is a very smart student. His attention is poor even when she talks to him directly. Speaking to his mother, she notes that at home he also seems very hyperactive, and makes decisions without really thinking about their consequences. He is always jumping around and breaking things. He is not organized and always seems to lose his notebooks and homework.

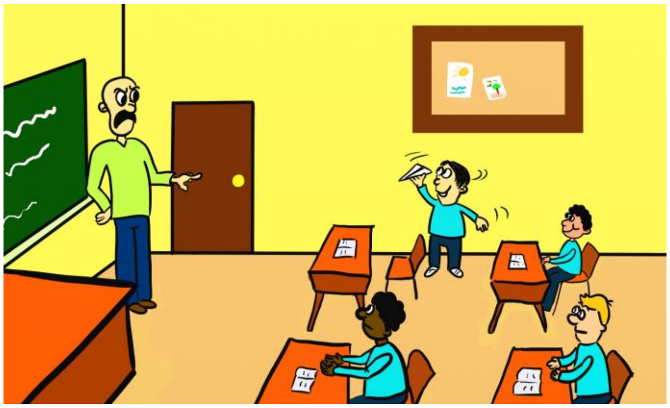

Questions to Consider:

What strategies could be implemented by the teacher to support Hassan?
What strategies could be implemented by the parent to support Hassan?

What strategies could be implemented by peers to support Hassan?

Who else could support Hassan and how?
When would you refer Hassan to a Specialist?

(A referral may be appropriate when the symptoms are disruptive to the classroom, impact the child’s educational attainment or that of others, put the child or others at a risk of injury and when classroom interventions are not sufficient to mitigate the problem).
