# Supplementary material for: Identifying pathways for large-scale implementation of a school-based mental health programme in the Eastern Mediterranean Region: a theory-driven approach
Source: Health Policy Plan. 2020 Nov 6;35(Suppl 2):ii112–23. doi: 10.1093/heapol/czaa124 (PMC7646738; doi:10.1093/heapol/czaa124)
Supplement: czaa124_Supplementary_Data [file czaa124_supplementary_data.zip › czaa124-suppl_data/Supplementary File 2.docx]

1. **ToC objective**
2. Describe the main objectives for the SMHP in your country as outlined in your ToC
3. **Assumptions and adaptation related to the implementation process**
4. Based on the ToC workshop or consultations in your country, describe **key assumptions** you have made (or intend to make) regarding the following process to facilitate the SMHP in your country?
   1. Planning
   2. Engaging
   3. Executing
   4. Evaluating
5. Based on the ToC workshop or consultations in your country, describe **key changes** you have made (or intend to make) regarding the following process to facilitate the SMHP in your country?
   1. Planning
   2. Engaging
   3. Executing
   4. Evaluating
6. **Contextual adaptation for SMHP**
7. Based on the ToC workshop or consultations in your country, describe **key assumptions** to the following dimensions you have made (intend to make) to facilitate implementation of the SMHP in your country*?*

- 1. Characteristics of individuals involved in implementing the SMHP (e.g. teachers in schools, parents, school health personnel, mental health service providers, district health managers etc.)
  2. Factors related to the organizations supporting the implementation of the SMHP (e.g. schools, district health department, MOH, Ministry of Education)
  3. SMHP program characteristics (the activity that will be used towards implementing the SMHP, including technologies that will be adopted by the organization/individual implementing the activities)
  4. Process of conducting the activities (how the activity will be implemented, including the planning, execution strategies, reflection and evaluation of activities, or adjustments made to the plan)

1. Based on the ToC workshop or consultations in your country, describe key assumptions to the following dimensions you have made (intend to make) to facilitate implementation of the SMHP in your country*?*
   1. Political environment (policymaker support, political climate accepting of SMHP, and political structure to conducive to coordinated action)
   2. Economic environment (sufficient revenue sources/base to fund activities and/or maintain system developments)
   3. Social environment (social norms around mental health and SMHP, accepting communities/families in which the SMHP will be implemented)

- 1. Technological environment (infrastructure or technological advances outside of the organization)
  2. Other environment (any other environment including the global policy environment and cross-organizational collaboration)

1. Based on the ToC workshop or consultations in your country, describe **key changes** to the following dimensions you have made (intend to make) to facilitate implementation of the SMHP in your country*?*

- 1. Characteristics of individuals involved in implementing the SMHP (e.g. teachers in schools, parents, school health personnel, mental health service providers, district health managers etc.)
  2. Factors related to the organizations supporting the implementation of the SMHP (e.g. schools, district health department, MOH, Ministry of Education)
  3. SMHP program characteristics (the activity that will be used towards implementing the SMHP, including technologies that will be adopted by the organization/individual implementing the activities)
  4. Process of conducting the activities (how the activity will be implemented, including the planning, execution strategies, reflection and evaluation of activities, or adjustments made to the plan)

1. Based on the ToC workshop or consultations in your country, describe key assumptions to the following dimensions you have made (intend to make) to facilitate implementation of the SMHP in your country*?*
   1. **Political environment** (policymaker support, political climate accepting of SMHP, and political structure to conducive to coordinated action)
   2. **Economic environment** (sufficient revenue sources/base to fund activities and/or maintain system developments)
   3. **Social environment** (social norms around mental health and SMHP, accepting communities/families in which the SMHP will be implemented)

- 1. **Technological environment** (infrastructure or technological advances outside of the organization)
  2. **Other environment** (any other environment including the global policy environment and cross-organizational collaboration)

1. **SMHP program adaptation**
